# Voluntarism as Resistance to State Control: A Case Study of the Kingston Victoria Hospital and the Fledgling NHS

**DOI:** 10.1093/shm/hkae034

**Published:** 2024-06-20

**Authors:** Steph Haydon

**Affiliations:** Third Sector Research Centre, School of Social Policy, Muirhead Tower, University of Birmingham, Edgbaston, Birmingham B15 2TT, UK

**Keywords:** National Health Service, patient participation, state control, voluntarism, cottage hospitals

## Abstract

With the launching of the National Health Service (NHS) in 1948, this taxpayer-funded, centralised, universal service seemingly negated the need for new voluntary hospitals to be established in Britain. Within 3 years, however, the former doctors of the Kingston and Malden Victoria Hospital (KMVH) announced a new voluntary hospital (the New Victoria) after the KMVH was closed for repurposing in the NHS. Examining this case reveals stakeholder perceptions of the early NHS, including debates over general practitioner (GP) independence, local democracy and state control which predated and permeated the founding of the Service. I argue the New Victoria was founded as a response to and revolt against centralised bureaucracy and an attempt to restore a sense of GP independence and patient control in the local hospital service. Voluntarism, in the form of a voluntary hospital, was the medium through which these debates took place.

With the advent of the National Health Service (NHS) in 1948, the vast majority of voluntary hospitals in Britain were taken into the new Service, to be funded and run by the state through general taxation rather than under the auspices of charitable effort.^1^ Charity and voluntarism would instead take a more marginal role in health provision, supplementing state-funded core services such as providing patient amenities and funding research.^2^ The need for new voluntary hospitals was seemingly over; the dawn of centralised, universal state-provided service had come.^3^ Yet, within 3 years, a new voluntary hospital was announced: the doctors of the Kingston and Malden Victoria Hospital (KMVH) announced they would be fundraising for a new voluntary—the New Victoria—after the KMVH had been repurposed under the NHS.^4^ This article examines the founding of the New Victoria, the first—and only—voluntary hospital established after 1948.

With the exception of Seaton,^5^ who used the case as a lens through which to examine the anti-NHS group the ‘Fellowship for Freedom in Medicine’ (FFM), the founding of the New Victoria has remained unexplored. The FFM allowed KMVH doctors to speak at their meetings, appointed some of the KMVH’s doctors as its first lay members,^6^ and reportedly part funded the campaign to save the KMVH.^7^ Seaton demonstrated how the case was cast by the FFM as an example of the ills of nationalised medicine. My analysis adds nuance to this account demonstrating how the attitudes of the KMVH’s doctors varied both over time and between public and private accounts. This paper also adds to the literature on post-1948 voluntarism, class, and patient participation in healthcare: the KMVH case reflects the different attitudes towards voluntarism in the health sector held by the Conservative and Labour parties, tensions between middle and working classes, and continued demand for patient participation which predate—and permeated—the founding of the NHS.

To fully establish the importance and meanings of this case, I first examine the post-war context in which the KMVH was repurposed and the New Victoria established, highlighting concerns from doctors, patients, and political parties over the expansion of state control that the NHS would entail. Second, I explore the case of the KMVH, investigating events and debates that took place between 1949, when the repurposing of the KMVH was announced, and 1958, when the New Victoria was opened. Through this, I identify and discuss two core themes: (i) the independence of GPs and the contribution of cottage hospitals; and (ii) patient control over hospital provision. I demonstrate that whilst the former was partially replaced within the confines of the NHS, the latter was portrayed by the KMVH’s doctors as only replaceable outside of the NHS. At no point did the KMVH’s doctors advocate specifically the benefits of voluntarism; voluntarism, in the form of a voluntary (cottage) hospital, was simply the vehicle through which debates over local versus centralised control took place. I therefore argue that the New Victoria was founded as an act of resistance to state bureaucracy and support for general practitioner (GP) independence, rather than an act of support or advocation for voluntary healthcare provision.

## Context: Post-war Concerns for State Control

Prior to 1948, healthcare in Britain was characterised by ‘welfare pluralism’^8^ comprising: a small private sector that catered to the upper classes, capable of paying for their healthcare; voluntary hospitals founded through charitable effort and staffed by honorary staff; and municipal hospitals—predominantly former poor law infirmaries which had been taken over by local authorities after the 1929 Local Government Act, and isolation and infectious disease hospitals.^9^ Provision was based on history, not rational assessment of need: the distribution of philanthropists to endow voluntary hospitals, and of local authorities which chose to enact their powers to take over poor law infirmaries, created irregular and uneven patterns of provision with varying rates of accessibility and utilisation.^10^ Notwithstanding issues in evaluating the connections between provision and need, it appears that the two rarely lined up.^11^

The establishment of the NHS in 1948 brought voluntary and municipal hospitals under the remit of the Minister of Health—then Aneurin Bevan (Labour Minister and NHS architect). By funding healthcare through general taxation, Bevan believed the inequity of healthcare distribution could be reduced. Most accounts of NHS history portray its formation as widely popular and politically ‘inevitable’ with near-universal public support.^12^ However, key tensions arose during the debate of the NHS Bill, as GPs’ independence and patient participation in the health service were seen as potentially vulnerable to state bureaucracy. The KMVH echoes these tensions, showing how they penetrated the NHS’ founding and played out in practice.

Prior to 1948, GPs had mostly worked out of their own surgeries and cottage hospitals and were accordingly afforded a high degree of autonomy.^13^ Cottage hospitals were typically small (averaging 15–25 beds),^14^ rural institutions catering to the village or small-town communities in which they were located.^15^ Like other voluntary hospitals, cottage hospitals were established through local charitable initiatives.^16^ They were an especially popular form of the hospital, favoured amongst patients and professionals alike for the doctor–patient proximity they afforded^17^; cottage hospitals were often referred to as GP hospitals as patients were attended to directly by their doctor.^18^ Cottage hospitals provided GPs with access to patients who they could court for their private practice, and considerable social standing within the community.^19^

Bevan’s proposals were considered a threat to GPs’ independence. The imposition of a state-run healthcare system was viewed by critics as totalitarian, placing the Minister of Health in a dictatorial position. In 1946, former chairman of the British Medical Association (BMA), Alfred Cox, even likened the proposals to Nazism: ‘I have examined the Bill and it looks to me uncommonly like the first step, and a big one, to national socialism as practised in Germany.’^20^ Using terms influenced by the recently concluded War, Bevan was cast as a ‘dictator’ and doctors in support of the NHS as ‘Quislings’ (a reference to the Nazi-appointed head of the Norwegian government during the country’s occupation).^21^ A key area of concern was over GP’s pay under the NHS: Bevan proposed to make GPs salaried employees; GPs wanted to remain independent with many threatening to boycott the service if the plans were to go ahead unchanged.

Fearing a loss in their autonomy and status, in January 1948, just 5 months before the launching of the NHS, 84 per cent of doctors voted against the Service in a vote held by the BMA.^22^ At the time, most doctors in Britain—and most members of the BMA—were GPs.^23^ To appease doctors, Bevan had to make various concessions, such as agreeing to retain GPs as independent contractors rather than employees. By the appointed day, most concerns appear to have been allayed as 86 per cent of GPs joined the NHS. However, several tensions remained: GPs complained of increased workloads in the early years of the NHS, particularly of excessive ‘paperwork’,^24^ and debates over pay remained largely unresolved until 1966.^25^ Amidst the trend towards medical specialisation, general practice was reportedly ‘in a state of crisis’, lacking morale and sense of professional identity, increasing the division between GPs and specialists.^26^ The FFM embodied these medical and ideological concerns, opposing what they saw as state interference in medicine. Founded in November 1948 by a group of conservative doctors, the FFM reached a height of more than 3,000 members and had the backing of several Members of Parliament (MPs) before its eventual demise in the mid-1970s.^27^ As highlighted by Seaton, the FFM served as the leading voice against the NHS in the 1950s.^28^ The intimate ties between the FFM and the KMVH are explored later in this paper.

Alongside these debates about GP independence in the new NHS, discussions over the future of patient participation also revolved around matters of control and perceived government over-stepping. Before the NHS was introduced, patients were able to exert a degree of control and influence in their medical provision through various individual and collective channels of participation. Private patients exercised ‘the power of the purse’, deciding which doctor to use and often seeking second opinions.^29^ In voluntary hospitals, significant donors were offered a lifetime governorship of the hospital,^30^ whilst subscribers were entitled to vote in general meetings and elect members of the managing committee.^31^ Committee membership was a source of social status and influence for the middle classes.^32^ Working-class representation was afforded through contributory schemes: individuals made small weekly contributions in return for access to hospital services without further payment.^33^ Contributory schemes grew such that at their peak they had some 10 million members and were credited with the financial survival of voluntary hospitals during the interwar period.^34^ Contributory fund committees had representation on hospital boards of management, typically selected from member-elected committees of the contributory scheme, who advocated for patient concerns.^35^

These channels of representation and control were heralded by contemporaries and more recent proponents as making services more responsive to the needs of the communities they served,^36^ embedding ‘grassroots democracy’ in healthcare.^37^ They were also considered sites of cross-class cooperation and solidarity, as middle and working classes were represented.^38^ This is somewhat idealised. In practice, subscribers were typically relatively small numbers of people, drawn from elite strata within communities: only those who could afford to subscribe were afforded the benefits therein.^39^ With the rise of contributory schemes, voluntary hospitals became less financially dependent on philanthropy,^40^ yet philanthropic elites continued to hold sway over working-class representatives on hospital boards. Working-class representatives were also typically over-ruled by medical professionals when the two groups were in disagreement.^41^ As summarised by Hayes: ‘not all hospitals welcomed a significant working-class membership on the boards of management, nor did increased representation reflect directly the scale of increasing worker financial contribution’.^42^ Committees still appeared to reproduce social hierarchies as leadership positions remained ‘monopolised’ by middle-class people,^43^ and working-class representation remained a minority (typically limited to a third of committee seats).^44^

Whatever their relative merits or shortcomings, under the NHS these direct channels of participation were removed and replaced with state-appointed Regional Hospital Boards (RHBs) and Hospital Management Committees (HMCs).^45^ Britain’s non-teaching hospitals were divided into 15 Regions, each under the control of an RHB, which were further sub-divided into Groups to be managed by a HMC.^46^ HMC members were appointed by their respective RHB committee who were themselves appointed by the Ministry of Health. The patient voice in the health sector was now indirect through the election of the government. Conservatives and contributory scheme representatives, amongst others, criticised this new system for taking control of hospitals away from patients and local people and placing it in the hands of ‘remote’ RHBs too distanced from the hospitals they were responsible for.^47^ The result is what has been called a ‘democratic deficit’ in the NHS.^48^

Past literature on patient participation has typically asserted or at least implied that after 1948, demand for public representation did not re-emerge in Britain until the appearance of patient groups and consumer bodies in the 1960s and 1970s.^49^ These associations engaged with health consumerism, advocating for patient rights and representation. Through examining the KMVH/New Victoria case, I demonstrate that demand for public participation and channels of local accountability in the NHS predates 1960s patient consumerism: the KMVH’s doctors and patients established a new voluntary hospital in the 1950s as a means of restoring pre-NHS models of patient participation.

It was against this backdrop of concerns for the future of general practice and shifts from local to centralised control that the KMVH was repurposed within the NHS, leading to the establishment of the New Victoria Hospital. The remainder of this paper thus examines these debates in relation to the KMVH/New Victoria case.

## 1896–1948: Beginnings of the KMVH

Both the KMVH and its eventual successor the New Victoria were established as cottage hospitals. Surrey (the county in which the KMVH and New Victoria were established) had a particularly strong history of cottage hospitals: the first recognised cottage hospital—Cranleigh Village Hospital—was established in Surrey,^50^ and at least 39 cottage hospitals were established here—more than any other county.^51^ The decision to first establish a cottage hospital in Kingston-upon-Thames was made at a public meeting held in 1896, attended by the town’s ‘burgesses’,^52^ to determine how to celebrate Queen Victoria’s Diamond Jubilee.^53^ The Hospital was subsequently built on land donated by the Duke of Cambridge (a member of the Royal family) who opened the hospital in December 1898.

Like other cottage hospitals, the KMVH relied heavily on voluntary contributions from individuals and organisations (e.g. local businesses, schools and churches). As the hospital had to compete with various other fundraising appeals in the community,^54^ the KMVH also relied on subscriptions (the KMVH had over 180 annual subscribers),^55^ investments and patient fees.^56^ From the hospital’s only surviving pre-1948 annual report, in 1928, the hospital had no contributions through contributory schemes—most income came from investment interest (38.8 per cent), from subscriptions and donations (gifts, collections, legacies) (29.9 per cent) and individual patient fees for services (28.4 per cent).^57^ These figures and the founding of the hospital suggest the KMVH was very much a middle-class institution: the hospital was located in an affluent borough and county dominated by the middle classes, its origins bore connections to the Royal family, and there is no evidence of working-class representation on the hospital’s committees.

## 1949–51: Planned Conversion in the NHS

On 5 July 1948, ownership of the KMVH was passed to the Ministry of Health as the hospital joined the NHS. The KMVH fell under the Kingston Group HMC within the South-West Metropolitan RHB. In May 1949, the South-West Metropolitan RHB sent a Review Committee to Kingston to evaluate hospital provision in the Group.^58^ The Review Committee determined the most pressing medical need in Kingston was increasing gynaecological and maternity beds.^59^ They subsequently recommended repurposing the KMVH from a cottage hospital into the gynaecological and maternity unit for the Kingston and District Hospital—a general hospital situated directly opposite the KMVH.^60^ The KMVH had been viewed favourably in terms of its staff, equipment and facilities in multiple visits from the General Nursing Council (GNC) and the King Edward’s Hospital Fund for London (King’s Fund).^61^ These assessments, combined with the ‘close physical proximity’^62^ of the KMVH and the District hospital made the planned merger appealing to the RHB as an efficient means through which to increase gynaecological and maternity provision.

However, when the Review Committee’s report was shared with the Kingston HMC in August 1949, the HMC’s Medical Advisory Committee rejected the proposed conversion of the KMVH ‘by a very narrow majority’.^63^ The RHB pressed on regardless. The proposed conversion was made public when the plans were leaked to, and reported by the town’s only local newspaper—the Surrey Comet—in September 1949.^64^ The RHB faced immediate and strong backlash from the KMVH’s doctors, all of whom were GPs. With support from their local Member of Parliament—Conservative John Boyd-Carpenter—the doctors appealed to the RHB to reconsider their proposal, to the public for support and to the Minister of Health to over-rule the RHB. Issued with a notice of closure (so the hospital could be converted) to take effect on 31 October 1950, the KMVH’s doctors announced a ‘stay in strike’^65^: the hospital doors were locked, and two ex-policemen formed a picket to check the identities of anyone attempting to enter the building.^66^ The doctors were reportedly unanimous in the decision to defy the closure notice: the Chairman of the KMVH House Committee, Brigadier Norman Skentelbery, stated ‘all of our 26 doctors are willing to go to prison if needs be’.^67^

Over the next 7 months, the KMVH’s doctors led a campaign against the Hospital’s conversion (discussed more below), featuring town hall meetings, a public petition, deputations with the RHB and Minister of Health, letters published in local and national newspapers, and a televised appeal. Despite these efforts, the KMVH was eventually closed for conversion on 31 May 1951. During this period (1949–51), arguments for preserving the KMVH as a GP hospital centred on three key topics—(i) patient–doctor proximity, (ii) homeliness and (iii) efficiency—which reflect debates raised during the reading of the NHS Bill.

(i) Patient–doctor proximity

The KMVH’s doctors argued the patient–doctor proximity characteristic of cottage hospitals enabled GPs to ‘keep in touch with matters medical’, upholding their interests and standards.^68^ Converting the KMVH from a GP hospital into a gynaecological ward was described as ‘the biggest nail in the coffin’ of General Practice.^69^ Closing cottage hospitals like the KMVH was described as leaving the GP ‘in medical isolation’,^70^ preventing them from keeping abreast of medical developments, and further eroding their responsibilities. Given the central role of the KMVH’s doctors in defending the hospital, I argue the campaign was motivated initially by a degree of professional interest—a desire to retain GP independence, control and relevance amidst the aforementioned ‘crisis’ in general practice and trend towards medical specialisation.^71^

Current and former patients of the KMVH similarly expressed their support for, and desire to retain, this doctor–patient proximity. Writing into the Surrey Comet, patients argued direct attendance by family doctors provided ‘peace of mind and trust’ which ‘often brings much courage and confidence to the patient’.^72^ Such assurance was presented as alleviating anxiety when going into hospital—for patients and their families—which contributed to a quicker recovery.^73^

The Hospital’s doctors and their political supporters deliberately related the KMVH to other cottage hospitals to attract wider public support and sympathy. For instance, in a televised campaign video, the KMVH’s doctors argued their campaign ‘does not only apply to our own Kingston Victoria Hospital but to many others across the country’.^74^ During a House of Commons debate on the future of the KMVH, the Conservative MP for Morecambe and Lonsdale similarly drew parallels with a hospital in his constituency before adding ‘There are also 20 or 30 other towns in which this situation exists.’^75^ Through such comparisons, the conversion of the KMVH was portrayed as part of a coordinated effort to eradicate cottage hospitals entirely and ‘keep the general practitioner out of the hospital service’.^76^

(ii) Homeliness

The KMVH was also defended on sentimental grounds as a homely, warm and welcoming institution in which patients felt comfortable.^77^ This was contrasted by doctors against perceptions of the far larger and ‘unwelcoming’ Kingston and District Hospital: ‘If this proposal is carried out it will only be a matter of time before similar action will be taken with hospitals at Surbiton, Thames Ditton, Teddington, Epsom, etc. All these, which were started with the help of the local practitioners and residents, and maintained by them, will be swallowed up by the great Clinical Concentration Camps that are proposed.’^78^ Comparisons to concentration camps so shortly after the conclusion of World War Two illustrate the strength of sentiment felt at the time. Such comparisons were made not only by the KMVH’s doctors but also by doctors at other cottage hospitals in Surrey concerned their hospital faced a similar fate.

The stark contrast between the KMVH and District Hospital conveyed appears to have stemmed from three underlying issues: the different sizes of, state of facilities at and origins of the two hospitals. First, whereas the KMVH was a small cottage hospital with 44 beds, the District Hospital was a much larger institution with 502 beds.^79^ The ‘homely’ atmosphere described by KMVH patients was deemed impossible to attain in larger hospitals,^80^ an opinion seemingly widely shared and supported by patient surveys from before 1948.^81^ Second, the state of facilities at both hospitals also varied: the KMVH was described in reports by the King’s Fund and the GNC as being well-equipped, bright and modern^82^; the District hospital was in need of repairs (from war damage)^83^ and updating.^84^ Finally, social attitudes towards each hospital’s origin likely also shaped opposition towards their merger: the KMVH was established as a voluntary hospital by the town’s burgesses on land donated by the Duke of Cambridge; the District Hospital was a former workhouse infirmary built by the local authority to care for the town’s ‘paupers’ under the English Poor Law. In Kingston-upon-Thames, a royal borough and one of the wealthiest areas in the country, classist snobbery towards a former Poor Law institution may have stimulated resentment towards the RHB’s proposed merger of the two hospitals. This reflects wider class differences in pre-1948 perceptions of the British hospital system highlighted by Hayes: middle-class people were more likely to have supported voluntary hospitals, and express concerns that the ‘human touch’ of voluntary hospitals would be lost in a ‘depersonalised’ state-run system; working-class people were generally more likely to prefer collectivist solutions to health, including state-run provision.^85^

Sentimental arguments over the ‘homeliness’ of the KMVH were criticised by Labour politicians. As noted by Labour MP Sir Frederick Messer: ‘Anybody at a town meeting could point to his little cottage hospital and describe it in terms that would wring tears from the eyes of stone statues. […] I plead with the Minister to let the regional boards get on with their job.’^86^ The leader of the local Labour party in Kingston similarly stated: ‘We are trying to hang on to a little bit of sentimentality which may be hindering the progress of real medicine and a proper hospital service’.^87^ Sentimentality, they argued, must not be allowed to impede efficiency.

(iii) Efficiency

The perceived efficiencies of cottage hospitals were a frequent point of debate during the reading of the NHS Bill. Bevan, for example, famously argued: ‘I would rather be kept alive in the efficient if cold altruism of a large hospital than expire in a gush of warm sympathy in a small one.’^88^ For Labour, the ‘homeliness’ of cottage hospitals came at the cost of hospital efficiency as they were too small to provide an array of specialist services.^89^ In contrast, Conservatives generally acknowledged possible inefficiencies but dismissed them as a tolerable price to pay.^90^

During the campaign to save the KMVH, supporters maintained the hospital operated with low costs and contributed to the efficiency of the wider health service. Writing to the Surrey Comet, one Kingston resident and husband of a former patient claimed: ‘The cost per bed is lower than in any other hospital within the Kingston Group, and the number of cases per bed higher than in larger hospitals’.^91^ The low cost-per-bed claims were reiterated by the KMVH’s doctors, but—due to lack of surviving data—cannot be verified. However, the claims made about cases per bed appear false: there were more cases per bed at the KMVH than at the District Hospital but fewer than Richmond and Surbiton which both had more beds, and fewer than Thames Ditton and Molesey which both had fewer beds.^92^ The KMVH’s daily occupation rate of 31 for its 44 beds was lower than nearly every hospital in the Group.^93^

Notably, no supporter of the KMVH (doctor, patient or politician) acknowledged any potential inefficiencies of the KMVH or cottage hospitals more broadly. Indeed, KMVH’s doctors argued that cottage hospitals contributed to an efficient NHS (primarily by alleviating pressure on beds) and so their removal would ‘harm’ the Service.^94^ In contrast, the RHB and their supporters highlighted efficiency issues. For example, Dr Stark Murray—a member of the Kingston HMC, President of the Surrey branch of the BMA and President of the Socialist Medical Association (1951–70)—argued that small GP-led units could not do ‘a modern hospital job’.^95^ The South-West Metropolitan RHB also noted that given the proximity of the KMVH to the District hospital, absorption of the former into the latter would increase efficiency by avoiding duplication of services.^96^

Across these areas of debate, views typically differed along political party lines. Labour politicians (both in the House of Commons and local Labour groups) backed the RHB’s proposal from the onset, arguing it would improve the scale and efficiency of hospital provision in the area for the majority of the local population.^97^ They dismissed the campaign against the KMVH’s conversion as a ‘Tory political stunt’ designed to reduce Labour support in the run-up to the general elections held in February 1950 and October 1951.^98^ With the exception of one Liberal MP, every politician who explicitly supported the KMVH’s doctors was a Conservative.

Political support for either side of the debate largely followed the corresponding party policies. The 1945 Conservative party manifesto was built upon visions of setting people free from wartime constraints.^99^ Labour, contrastingly sought to build upon and in many ways extend state control with an extensive programme of nationalisation including not just health but also finance, iron and steel and transportation. Though the Conservatives voted against the second and third readings of the NHS Bill, by the 1950 general election, the Conservatives had largely accepted the NHS and did not seek to reverse it—instead pledging to ‘maintain and improve the Health Service’—but did express explicit support for ‘the position of the family doctor by restoring his freedom to practise anywhere and by offering a weighted capitation fee to doctors with small lists, especially in rural areas’.^100^ The Conservatives prioritised narratives of individual freedoms and decentralisation of power; Labour emphasised collective progress, community and cooperation.^101^

The KMVH’s doctors and the politicians who supported them reflected this shift in Conservative policy: though Kingston MP Boyd-Carpenter had, along with the rest of the Conservative party, voted against the NHS and other nationalisation efforts since the 1945 election, he and the KMVH’s doctors publicly maintained their campaign was non-political and that they only wished to see the NHS ‘preserved and extended’.^102^ These supporters of the KMVH were concerned with the retention of their cottage hospital within the NHS, rather than opposition to the NHS or state-provided healthcare more broadly.

## The Claremont Hospital: Replacing GP Beds

Because of the outrage stirred by the plans to convert the KMVH and the portrayed threat to GP beds, the RHB offered the KMVH's GPs the Surbiton Annexe as a replacement. Originally a cottage hospital in Surbiton (one mile away from the KMVH), the Surbiton Annexe had been purchased by the Kingston and District Hospital in 1935 to accommodate chronic patients.^103^ More than £6,000 was spent converting the Annexe between 1950 and 1951, restoring it to a GP hospital.^104^ It opened as ‘Claremont Hospital’ on 31 May 1951—the same day the KMVH was closed for conversion.^105^

The KMVH’s doctors protested the Annexe was too small and inadequate, opening with 28 beds compared to the KMVH’s 44. Even an expenditure of £6,000, they argued, would ‘fail to convert the sow’s ear into a silk purse’.^106^ These criticisms seem to have been justified given later evaluations of the Claremont. A review from the GNC in February 1952 reported issues with Claremont’s layout including narrow corridors and isolated positioning of two wards,^107^ and a King’s Fund report from 1954 questioned whether the hospital was even necessary.^108^ Despite these objections, the Claremont was accepted by 140 GPs in the Kingston Group and then Minister of Health Hilary Marquand described it as a ‘reasonable alternative’ to the KMVH.^109^ The 26 KMVH doctors remained unsatisfied.

## 1951–58: Establishing the New Victoria

On 1 June 1951, the day following the closure of the KMVH (and the opening of Claremont Hospital), the former KMVH doctors announced at a press conference the formation of a charitable trust—the Kingston and Malden Victoria Medical Foundation. Brigadier Skentelbery was the Foundation’s first President, and former KMVH doctor Dr Bernard Lake was the first Chairman. The Foundation was intended to fundraise £40–50,000—the amount calculated as needed to establish and run a new voluntary hospital for at least 2 years.^110^ The appeal for funds was shared not only with local residents, but also with every GP in Britain. As argued by Dr Lake: ‘although the immediate issue is a local one, the implications of the scheme may affect doctors everywhere’.^111^

Over the next 7 years, £35,000 was raised from 5,000 contributors through public donations and subscriptions of three pence per week.^112^ By October 1954, enough funds had been raised to purchase a property—Coombe Manor—on the same lane that the KMVH had been situated on.^113^ Following the development of the property, the New Victoria was opened by the Duchess of Sutherland in May 1958.^114^ It was the first voluntary hospital founded since the NHS was established.

During the dispute over the fate of the KMVH, the hospital’s doctors painted the hospital’s conversion as part of an attack on GPs and cottage hospitals. Their campaign aimed to retain the KMVH within the NHS. During this period, there was no sense that the KMVH as a cottage hospital was antithetical to a free-at-the-point-of-delivery, tax-funded, national system. Quite the opposite—cottage hospitals were considered a vital element of an efficient NHS. Only once it was clear the conversion of the KMVH would go ahead did the doctors push to replace the hospital outside the system. These doctors, and their continued political supporters, argued the most important thing that had been lost through the process of the KMVH’s conversion was a sense of democracy. As noted by the vice-Chairman of Surrey Liberal Organisation: ‘Quite apart from the pros and cons regarding the immediate future use of the hospital, I feel convinced that the overriding factor is a grave infringement of the democratic rights of individuals and groups of individuals.’^115^

Whilst the subsequent effort to establish the New Victoria still centred on restoring GP autonomy, unsatisfied by the Claremont offering, the campaign narrative also focussed on restoring a sense of local control over hospital provision after feeling thwarted by NHS bureaucracy. A new cottage hospital set up as a voluntary outside the NHS was not advocated as a better type of healthcare but as the only viable option for reinstating local public and patient control over their healthcare provision. Throughout the dispute regarding the KMVH (i.e. 1949–51), the RHB was criticised for (i) going against local public opinion; (ii) being unrepresentative of local public opinion; and (iii) refusing to hear local public opinion. Such concerns similarly reflect middle-class opinion from before 1948: ‘The middle classes, too, just as they more strongly favoured voluntary hospital provision, were equally resistant to officialdom and intrusive state interference’.^116^

(i) Going against local public opinion

First, the RHB was portrayed by its doctors and political supporters as going against near-total consensus in the Kingston and neighbouring Malden and Wimbledon communities. As summarised by the Conservative MP for Wimbledon Cyril Black: ‘we have here almost the position of the Regional Board versus the rest of the world, so strong, so unanimous and so complete is the objection on the part of all other sections of the public’.^117^ The most significant evidence supporting this claim is the public petition delivered to the Minister of Health in February 1951. The petition appealed: ‘that this retrograde step in the development of the National Health Service should be remedied by general practitioner hospitals, such as the Kingston and Malden Victoria Hospital, being established as an essential factor in the Service’.^118^ The petition was signed by 40,780 people^119^; the combined adult population of Kingston and Malden was roughly 50,000 at the time.^120^ Notably, Labour had advised its members in Kingston and Malden not to sign the petition.

In November 1950, some 2,000 people attended a public meeting about the proposed conversion of the Hospital. 1,500 people attended inside—more than 300 people over the venue’s capacity.^121^ A further 500 people attended outside the venue where the speeches from local MPs and Mayors were broadcast via speakers.^122^ According to the Surrey Comet, it was the largest meeting held in Kingston’s history and the overwhelming majority opposed the KMVH’s conversion.^123^ Labour representatives who spoke in support of the RHB were reportedly ‘drowned out’.^124^

Various local councils and committees also backed the campaign to maintain the KMVH as a GP hospital. Both the Kingston and Malden Conservative-run town councils made official appeals to the RHB and Minister of Health to at least delay, if not repeal, the decision.^125^ Additionally, the Conservative MPs for Kingston (a constituency which included Malden) and neighbouring Wimbledon both supported the campaign in Parliament, appealing directly to the Minister of Health on several occasions, including a personal deputation. The campaign against the conversion of the KMVH was also officially and unanimously supported by various small groups in the area including the Kingston Chamber of Commerce (representing 550 local businesses),^126^ the Malden Rotary Club,^127^ the Kingston Public Health Committee,^128^ and even the Malden United Housewives Association,^129^ all of which were largely middle-class institutions.

Given this widespread support, the KMVH’s doctors, local political representatives and even the BMA accused the RHB of ‘flouting public opinion in the district’.^130^ When Kingston MP Boyd-Carpenter raised this matter in Parliament he was told it would be ‘inappropriate’ for the Minister of Health to intervene. Boyd-Carpenter subsequently criticised the ‘apparent immunity to democratic process with which regional hospital boards were endowed’.^131^ Notably, this ‘immunity’ was a result of political compromises made during the establishment of the NHS to insulate the medical profession from local authority control. As recalled by Labour MP Messer, during a debate on the KMVH: ‘when the Act, as a Bill, was debated, the Opposition [the Conservatives] had insisted that there should be no interference from Whitehall’.^132^ Calls for overturning or intervening in the RHB’s decision on the KMVH were therefore dismissed as hypocritical exceptionalism.

(ii) Being unrepresentative of local public opinion

Second, the KMVH’s doctors and their supporters portrayed the RHB as being unrepresentative of the local public and GPs. Given the South-West Metropolitan RHB—an unelected body—consistently rejected the appeals of elected representatives (e.g. town councils, Mayors, MPs) and did so without any GPs on the Board, the RHB was criticised as ‘undemocratic’,^133^ ‘remote from the practice of medicine’ and ‘remote from the views of the individual patients’.^134^ Doctors—both at the KMVH and other GP hospitals—deplored the ability of this non-elected board to act against the wishes of such an overwhelming majority of the local community. As argued by one critic when writing to *The Times*: ‘the service has set up large corporations, in this case, the regional hospital boards, which administer the hospitals over a wide area according to the well known traditions of a nationalized industry, i.e., with little or no regard to the wishes of the consumers – in this case the patients.’^135^ These strongly echo concerns raised during readings of the NHS Bill regarding the transfer of control from local patients to remote government-appointed boards.^136^

Health authorities countered that the KMVH did not itself represent the medical needs of the majority of the community: ‘Who are deserving of the greatest consideration – the women resident in our area who are sorely in need of these extra gynaecological beds, or whether a small minority should continue to enjoy a special advantage, if indeed it be a special advantage, of receiving treatment at the Victoria Hospital?’.^137^ Given only a minority (5 per cent) of patients in Kingston were treated at the KMVH, the RHB and their Labour party supporters argued this minority should not be allowed to continue their ‘special privilege’ at the cost of a larger number of patients with more pressing needs.^138^

Debates over the representativeness of the RHB were amplified when several members of the Kingston HMC were not reappointed to the Committee. When the proposed conversion of the KMVH was first revealed in 1949, the Kingston HMC voted against the proposal, calling for delay and reconsideration. In March 1950, during regular HMC appointments, the RHB did not reappoint four of the HMC members who had been most critical of the proposals, including the Committee Chair.^139^ These were replaced with new members who were more amenable to the RHB’s plans, including a Chairman who explicitly stated he did not believe the Committee had the power to stand against the RHB.^140^ This new HMC voted to accept the RHB’s proposal in December 1950.

The non-reappointment of these Committee members was described by the KMVH’s doctors and their political supporters as ‘totalitarian’,^141^ ‘dictatorial’,^142^ ‘Hitler methods’ and a ‘violent attack’^143^ aimed at ‘beheading’ the opposition.^144^ Such an ‘attack’, critics argued, was a threat to democracy. As summarised by Skentelbery: ‘What I am most anxious and most incensed about is the fact that a board can pay lip service to democracy and depart from it as soon as ever it is opposed’.^145^ To address these concerns, Skentelbery argued RHBs should be elected in order to embed local opinion: ‘The whole thing should grow from the bottom, the public choosing the house committees, the hospital choosing their representatives and the latter choosing representatives for the Board’.^146^ Such views are based on an idealised recollection of pre-1948 hospital management—as outlined above, pre-1948 mechanisms were not wholly representative or democratic. Furthermore, Bevan had initially favoured a more localised model of the NHS but had been thwarted by the BMA’s opposition. As noted by Daunton, the 1948 founding structure of the NHS was not so much an outcome of Labour’s commitment to such structures as it was of the medical profession’s resistance to alternatives: ‘Multipurpose local government was more of a threat to their position than nationalization’.^147^

Responding to these criticisms, the RHB argued their decision bore no relation to the KMVH dispute. Instead, they maintained, they were guided by a recent ministry circular that advocated the potential benefits of appointing new members to HMCs.^148^ The RHB reported that they had been ‘dissatisfied with the Kingston Hospital Management Committee whose administration has not been particularly satisfactory or economical’.^149^ In particular, the RHB criticised the lack of attendance of some of the replaced Committee members,^150^ and saw advantages in having a Chairman who was ‘not directly connected with the local affairs of the Group and thereby able more easily to adjudicate on any contentious matter.’^151^

It is notable that Kingston was not unique in such complaints over HMC appointments: during the initial establishment of HMCs, Bevan received hundreds of letters from various hospitals, political and religious groups and councils complaining about their perceived lack of representation.^152^ All were informed in response that HMC appointments were a matter for the RHB and not the Minister. Likewise, when HMC members were not reappointed to their positions, they had often also written to the Minister to complain^153^; it was not unique either for members to not be reappointed, or for those members and/or their supporters to voice their complaints to the Minister of Health.

(iii) Refusing to hear local public opinion

Third, the RHB was criticised for withholding key discussions and plans from the public and refusing to hear public opinion. The initial proposals for the conversion of the KMVH were revealed to the public by the press, not the RHB, and after objections were raised, the RHB repeatedly voted to discuss the matter of the KMVH without the press present, later sharing only concise statements. This ‘scheming’ and ‘secretive’ approach was condemned by the KMVH’s doctors and their supporters for lacking transparency and impeding accountability.^154^

To address concerns over the RHB’s actions, multiple calls for a public inquiry were made. As justified by MP Boyd-Carpenter: ‘one of the major reasons why local opinion felt that an injustice had been done was that no opportunity had yet been given for local opinion to express itself directly to those responsible’.^155^ In March 1950, Boyd-Carpenter called for an inquiry into the RHB’s dismissal of members of the Kingston Group HMC.^156^ In November 1950, the BMA called for an inquiry into the RHB’s proposals to convert the KMVH^157^—a call subsequently repeated by the KMVH’s doctors^158^ and the MPs for Kingston and Wimbledon on several occasions each.^159^

These appeals were rejected by the Minister of Health and Labour MPs who believed agreeing to an inquiry would set a precedent meaning no RHB could make any decision without ‘looking over their shoulders to see whether what they were doing met with the approval of the Minister’.^160^ According to one Labour MP, agreeing to an appeal would lead to the ‘breakdown of the service’.^161^ Both Bevan and his successor as Minister of Health Marquand repeatedly refused calls for inquiry: Bevan proclaimed the House of Commons was the ultimate ‘court of inquiry’^162^ and Marquand similarly maintained that through discussion in the House of Commons, there had already been ‘ample inquiry’.^163^

The narrative campaigners created of the RHB refusing to hear public opinion is rather misconstrued. Not only was the future of the KMVH debated several times in the House of Commons, but the hospital’s doctors and their political supporters also had separate deputations with the RHB^164^ and the Minister of Health,^165^ and were invited to offer alternative proposals to the RHB.^166^ Following a meeting between the RHB, HMC and two of KMVH’s doctors in December 1949, the HMC was invited to submit alternative plans for providing the gynaecological and maternity beds needed in the Group.^167^ A sub-committee was established, which included the two KMVH doctors who had been present at the meeting. The sub-committee’s recommendation of building a £25,000 extension to the general hospital^168^ was rejected by the RHB as too costly.^169^ In March 1951, several of the KMVH’s doctors and Boyd-Carpenter also had a deputation with then Minister of Health Marquand. Although the final decision was not what the KMVH’s doctors and their supporters wanted to hear, their voices had certainly been heard.

When Marquand upheld the RHB’s decision to convert the KMVH, and declined for the final time to hold a public inquiry, the KMVH’s medical staff and house committee issued the following response: ‘Our attitude remains what it has always been. We know we are right – in the interests of our patients and the wider interest of the public, as against a blind and bigoted new bureaucracy – and we maintain our stand until ejected.’^170^ The KMVH’s doctors subsequently held a meeting—records of which have not been kept—to determine their next action. It was after this meeting, and the closure of the KMVH, that the plans for establishing the New Victoria were announced.

## The New Victoria Hospital: Reinstating Local Control

To restore the perceived loss of voice in and control over their local hospital provision, the former KMVH doctors and Kingston and Malden residents who supported their cause subscribed to and backed the establishment of the New Victoria. Amidst a context of concern for state control and in an affluent, predominantly middle-class county dominated by Conservative politics, voluntarism—in the form of a new voluntary hospital—was portrayed as a vehicle for local democracy, an escape from ‘the paternal embrace’ of the NHS.^171^ The New Victoria’s founders tried to restore a sense of local control by implementing pre-NHS methods of patient participation and accountability. At no point did the KMVH’s doctors advocate specifically the benefits of voluntarism, or describe efforts to address pre-NHS limitations of voluntary hospitals. Their goal was to restore local patient and public control, not advance voluntary action.

The establishment of the New Victoria was heralded as a victory for democracy. Outside of and independent from the apparent ‘bureaucratic’ confines of the ‘dictatorial’ RHB, the New Victoria liaised openly with the local community from the onset, sharing information transparently. Whereas the RHB had held meetings about the Hospital ‘in secret’, the Foundation held AGMs at which ‘[a]ny member of the public who is interested in the New Victoria Hospital is welcome to attend’.^172^ These meetings were often attended by the Mayors of Malden and Kingston.^173^ Whilst the RHB had withheld key decisions from the public and press, Kingston and Malden residents were regularly updated on the progress of the new hospital through the publication of *The New Victoria: A Quarterly Magazine of News, Views and Events of The Kingston & Malden Victoria Medical Foundation* and Annual Reports stating income and expenditure, patient statistics and governance details (e.g. staff and committee members). These were both shared for free with local residents who were encouraged to share their copies with others.^174^

Local voice was also included in the foundation and running of the New Victoria. Mirroring how the original KMVH had been run prior to the NHS, the New Victoria was managed by an elected Board of Management and Board of Governors. Any individual or organisation who donated 50 guineas or more in a single year was offered a ‘Life Governorship’ of the Hospital. At least 17 Local Area Committees were established, each of which had an elected Chair and collectively had over 800 members.^175^ These Committees primarily fundraised for the new hospital but were also represented on the Boards of Management and Governors, giving them ‘a voice in the effective running of the hospital’.^176^ Though one can question how representative these Boards and Committees were of the 50,000 residents of Kingston and Malden, the New Victoria’s supporters evidently believed they were more representative than the 15 members of the RHB-appointed HMC they had previously dealt with. Dr Lake expressed his hope that such endeavours at the New Victoria ‘might even initiate administrative reforms that would make a healthier Health Service everywhere’.^177^

Establishing the New Victoria was presented as restoring the local community’s and doctors’ sense of ownership of ‘their’ hospital. Through the conversion of the KMVH, the hospital’s former doctors, along with Kingston and Malden residents, had lost a local resource over which they had perceived a sense of ownership and autonomy.^178^ Throughout the dispute, patients, doctors and local residents had referred to the KMVH as ‘their’ hospital,^179^ condemning ‘the destruction of the hospital built by and for local people’.^180^ Before the NHS, the very idea to build the hospital had been proposed by the community, and it was built and sustained over 50 years through their contributions—financial and otherwise.^181^ This sense of local ownership was not replaced by the offer and conversion of the Surbiton Annexe—the move was criticised by Boyd-Carpenter as ‘a transmigration of souls’^182^ and an effort aimed ‘utterly to destroy the identity of the Victoria Hospital’.^183^ The feeling of ownership was however restored through the establishment of the New Victoria which, upon its opening, was described as the ‘Town’s own hospital’.^184^ This is an interesting contrast against the early promotion of the NHS in 1948 as ‘Your National Health Service’.^185^

Such comparisons of ‘our hospital’ against ‘our NHS’ illuminate the different conceptions of ‘public’ conveyed throughout this case. The KMVH/New Victoria doctors and their Conservative supporters prioritised a ‘local public’ comprised of Kingston and Malden residents, predominantly those who were their patients. Thus, despite multiple deputations between doctors, politicians and policymakers during the KMVH dispute, they persisted ‘public’ voice had still not been heard, and, therefore, local residents were afforded channels of representation in the New Victoria. Labour prioritised a ‘national public’, represented through government and served by a national system.

This portrayal of the New Victoria as a democratic haven resonated with the medical conservativism of the FFM. In his analysis of the FFM, Seaton demonstrated the Fellowship’s medical, economic and ideological opposition towards nationalised medicine. The FFM saw the NHS as ‘an economically dangerous bureaucratic machine that crushed medical independence and risked pushing the country towards dictatorship’.^186^ The FFM hence embraced the KMVH dispute as a poster boy for their anti-NHS cause, portraying it as evidence against the ‘evils’ of bureaucracy and state control:

The health of the nation was controlled by a bureaucracy … . [T]he Minister of Health was a dictator, under whom the service was controlled downwards. Such a system destroyed the freedom of the individual and disregarded the collective wishes of the people.^187^

However, the KMVH’s doctors’ own views of the NHS—and of the FFM’s opposition to it—are decidedly more complex.

Before the KMVH was closed for conversion, none of its doctors aligned themselves publicly with anti-NHS views. The defence of the KMVH was a defence of GPs in hospitals, driven by the professional interests of the KMVH’s own doctors. When the KMVH’s Dr Tom Morgan spoke at the FFM’s annual general meeting (AGM) in 1950, his speech focussed on the interests of the General Practitioner—thus resonating with the medical concerns of the FFM—but did not raise any economic or ideological opposition to the NHS. Privately, some of the KMVH’s doctors may have supported the FFM’s ideological stance: when Skentelbery was elected the FFM’s first lay member in 1950, his wife (also a doctor) was already a regular member.^188^ However, publicly at this time, the KMVH’s doctors continued to insist that their efforts aligned with the values of the NHS. This suggests, that whilst they welcomed the FFM’s support, they largely—and certainly not publicly—did not share the FFM’s ideological opposition.

After the KMVH was closed for conversion, this view seems to have transitioned and connections with the FFM strengthened. The FFM hosted an ‘in memoriam’ event the day the KMVH was closed for conversion, attended by the KMVH’s doctors.^189^ Dr Lake subsequently adopted similar language to the FFM when campaigning for the New Victoria, describing the NHS as a ‘machine’ that regarded patients as ‘units’ rather than people.^190^ The FFM founder—Lord Horder—volunteered as an honorary consulting physician of the New Victoria Hospital,^191^ and then FFM Chair Dr Hale-White was invited to speak at the Victoria Foundation’s AGM in 1961, indicating at least some resonance with the doctors’ own opinions. Speaking at the AGM, Dr Hale-White celebrated the New Victoria as a ‘bulwark of freedom against state monopoly’ which ‘shines as an example of enterprise across a flat and uninteresting sea of control’.^192^

Yet, in contrast to the FFM’s unwavering opposition to the NHS, the New Victoria was still not portrayed by its doctors or fundraising committees as against the NHS in principle. In a promotional leaflet for the New Victoria, the Foundation explicitly stated as such:

Is the new Hospital in any sense in opposition to the National Health Service? Emphatically NO. *The exact opposite is the case*. The NEW Victoria Hospital will supplement and help (by providing additional accommodation and facilities) the services provided by the State.^193^ [all emphasis in original]

Whilst this statement was likely an effort to gain non-partisan support and funds for their campaign, it directly conflicts with the FFM’s opposition to the NHS. Whereas the FFM argued that private practice was needed to avoid ‘State monopoly of medicine’,^194^ the New Victoria’s staff presented themselves as supporting the state system.

## After 1958: Barriers to Voluntarism?

The Foundation’s initial funding proposals for the New Victoria were to raise £40–50,000 in donations and subscriptions to fund the Hospital’s first 2 years. Thereafter, the income from two private beds was expected to cover most of the projected £13,000 annual running costs of the hospital, allowing minimum reliance on voluntary contributions.^195^ Not only did initial funds raised fall short of their target, and take years longer than hoped, but running costs also soon exceeded these expectations and sufficient financial support was often challenging to attain.

After the appeal launched in 1951, the Foundation solicited support from The King’s Fund. However, these appeals were repeatedly rejected as the Fund’s Management Committee believed ‘that the hospital wanted a grant not so much for the money as for the prestige that would be conferred if the Fund implied approval’.^196^ It was not until the RHB expressed a will to ‘bury the hatchet’,^197^ that the King’s Fund were willing to support the New Victoria.^198^ The King’s Fund contributed £1,000 in 1960 to various maintenance and infrastructure costs,^199^ and a further £4,000 in 1962 covering most of the costs of the Hospital’s new operating theatre.^200^ In 1960, the New Victoria was described as ‘trying to get contractual relations with the Board’,^201^ although there are no records from the RHB indicating these efforts were successful.

By 1966 the hospital had an annual shortfall of £4,000,^202^ and in 1973 an appeal was launched for £200,000 to build ‘a 16-bed private wing to help with the financial support of the rest of the hospital’s resources’.^203^ This gradual trajectory towards private medicine reflects fundraising challenges experienced by voluntary hospitals before 1948 which had led to their reliance on patient fees and contributory schemes. The pre-1948 healthcare model adopted by the New Victoria struggled in the post-1948 context. Voluntarism had not wholly been eliminated under the NHS—nor had that been Labour’s aim—but the main forms of voluntary action in the health sector did shift significantly. Labour opposed Victorian, paternalistic charity, the type which had founded and endowed hospitals prior to 1948. However, they supported mutualistic voluntary efforts.^204^ Though NHS hospitals could still receive donated funds,^205^ most voluntary actions in the health sector shifted away from financial contributions and towards acts of service.^206^ Critics of the KMVH’s doctors similarly criticised the New Victoria’s establishment as a ‘retrograde step’^207^ in healthcare: ‘though private money may be forthcoming for isolated endeavours of this sort, it will never again be found for a comprehensive hospital service’.^208^

## Conclusions

The New Victoria’s founding is far more than an example of anti-NHS sentiment. Beginning as a defence of GP independence, the ensuing debates reflected pre-1948 tensions between the middle and working classes and between Conservative and Labour ideologies. They also highlight the persisting demand for patient input in healthcare.

Before 1948, middle-class people were more likely to support voluntary hospitals, oppose state interference, and, after 1948, to complain about the NHS.^209^ The campaign to establish the New Victoria appealed to these middle-class preferences, and, like the KMVH before it, mostly served and was supported by middle-class patients and locals. Ultimately, it therefore faced the same financial difficulties that the KMVH and voluntary hospitals had faced prior to 1948: after the initial excitement around the campaign had died down, voluntary contributions were not sufficiently forthcoming to sustain the hospital. Without a contributory scheme or core funding from the state, New Victoria had to expand private provision as a means of survival.

The New Victoria case additionally reflects Conservative-Labour differences during the 1950s in terms of attitudes towards nationalisation, voluntarism and conceptions of ‘the public’. The Conservatives’ prioritised individual freedom and responsibility, supporting the independence of medical practitioners and local patient voices against state control; Labour prioritised centralised control with accountability through government, and an efficient healthcare system for the national population. Conservative politicians thus backed the KMVH’s doctors; Labour backed the RHB.

The founding of the New Victoria also demonstrates that concerns for patient participation in healthcare did not simply disappear in 1948, only to later re-emerge in the patient consumerism movement of the 1960s. Rather, the debates over democracy which predated the establishment of the NHS^210^ permeated the founding of the service and were accentuated during the debate over the future of the KMVH. Some even used the language of consumerism in the 1950s,^211^ and expressed hope their actions would initiate channels of patient and public participation in the NHS itself.^212^ Whilst the KMVH’s doctors were unique in setting up a voluntary hospital to address their concerns, they were not alone in their objections: calls for improved representation of HMCs continued for several years across the NHS.^213^ It appears demand for patient participation in the NHS is as old as the NHS itself.

## Footnotes

1 Rudolf Klein, *The New Politics of the NHS: From Creation to Reinvention* (Oxford: Radcliffe Publishing, 2006[1983]); Charles Webster, *The National Health Service: A Political History* (Oxford: Oxford University Press, 2002[1998]).

2 British Hospitals Association, ‘Personal Support for Hospitals in the National Health Service: Is It Needed? What Form Should It Take?’, in *The Hospitals Yearbook, 1948* (British Hospitals Association, 1948).

3 Bernard Harris and Rosemary Creswell, ‘The Legacy of Voluntarism: Charitable Funding in the Early NHS’, *Economic History Review*, 2023.

4 ‘£50,000 Appeal for New Victoria’, *Surrey Comet*, 2 June 1951, 5.

5 Andrew Seaton, ‘Against the “Sacred Cow”: NHS Opposition and the Fellowship for Freedom in Medicine, 1948–72’, *Twentieth Century British History*, 2014, 26, 424–49.

6 ‘Kingston Victoria Hospital’, *Supplement to the British Medical Journal*.

7 Seaton, ‘Against the Sacred Cow’.

8 G. Finlayson, Citizen, State and Social Welfare in Britain, 1830–1990 (Oxford: Oxford University Press, 1994).

9 Martin Gorsky, ‘Community Involvement in Hospital Governance in Britain: Evidence from Before the National Health Service’, *International Journal of Health Services*, 2008, 38, 751–71.

10 Martin Gorsky, John Mohan and Martin Powell, ‘British Voluntary Hospitals 1871–1983: The Geography of Provision and Utilization’, *Journal of Historical Geography*, 1999, 25, 463–82.

11 John Mohan and Martin Gorsky, *Don’t Look Back? Voluntary and Charitable Finance of Hospitals in Britain, Past and Present* (London: Office of Health Economics and Association of Chartered Certified Accountants, 2001).

12 Klein, *The New Politics of the NHS*, 1–2.

13 Stevens, *Medical Practice in Modern England.*

14 H. C. Burdett, *Cottage Hospitals, Fever and Convalescent* (London: The Scientific Press Ltd, 1896).

15 McConaghey, ‘The Evolution of the Cottage Hospital’.

16 Hall, ‘From Cottage to Community Hospitals’.

17 Nick Hayes, ‘Did We Really Want a National Health Service? Hospitals, Patients and Public Opinions Before 1948’, *English Historical Review*, 2012, CXXVII, 621–61.

18 John Mohan, *Planning, Markets and Hospitals* (London: Routledge, 2002).

19 Hayes, ‘Did We Really Want a National Health Service?’.

20 Quoted in: Anthony Broxton, ‘“Why Should the People Wait Any Longer?” How Labour Built the NHS’, *London School of Economics*, 2017, https://blogs.lse.ac.uk/politicsandpolicy/why-should-the-people-wait-any-longer-how-labour-built-the-nhs/, paragraph 10.

21 Elston Grey-Turner and F. M. Sutherland, *History of the British Medical Association: Volume II, 1932–1981* (London: BMA, 1982), 64.

22 David Morrell, ‘Introduction and Overview’, in Irvine Loudon, John Horder and Charles Webster, eds, *General Practice Under the National Health Service 1948–1997* (London: Clarendon Press, 1998), 1–6.

23 Rosemary Stevens, *Medical Practice in Modern England: The Impact of Specialization and State Medicine* (Transaction Publishers, 2003), 153–68.

24 Gareth Millward, ‘Sick Notes Are a Waste of Time: Doctors’ Labour and Medical Certification at the Birth of the National Health Service’, in Jennifer Crange and Jane Hand, eds, *Posters, Protests, and Prescriptions: Cultural Histories of the National Health Service in Britain* (Manchester: Manchester University Press, 2022), 54–75.

25 Charles Webster, ‘The BMA and the NHS’, *British Medical Journal*, 1998, 317, 45–47.

26
*Ibid.*; Stevens, *Medical Practice in Modern England*; Seaton, ‘Against the Sacred Cow’.

27 Seaton, ‘Against the Sacred Cow’.

28
*Ibid*.

29 Alex Mold, ‘The Art of Medicine: Making British Patients into Consumers’, *The Lancet*, 2015, 385, 1286–87.

30 Anne Borsay, ‘“Persons of Honour and Reputation”: The Voluntary Hospital in an Age of Corruption’, *Medical History*, 1991, 35, 281–94.

31 R. M. S. McConaghey, ‘The Evolution of the Cottage Hospital’, *Medical History*, 1967, 11, 128–40; John Hall, ‘From Cottage to Community Hospitals: Watlington Cottage Hospital and Its Regional Context, 1874–2000’, *Local Population Studies*, 2012, 88, 33–49.

32 Martin Daunton, ‘Payment and Participation: Welfare and State-Formation in Britain 1900–1951’, *Past & Present*, 1996, 150, 169–216.

33 Martin Gorsky, John Mohan and Ted Willis, ‘Hospital Contributory Schemes and the NHS Debates 1937–46: The Rejection of Social Insurance in the British Welfare State?’, *Twentieth Century British History*, 2005, 16, 170–92.

34
*Ibid*.

35 Alex Mold, ‘Patient Groups and the Construction of the Patient-Consumer in Britain’, *Journal of Social Policy*, 2010, 39, 505–21.

36 Arthur Seldon, ed, *The Litmus Papers: A National Health Dis-Service* (London: Center for Policy Studies, 1980); Hayes, ‘Did We Really Want a National Health Service?’.

37 Gorsky, Mohan and Willis, ‘Hospital Contributory Schemes and the NHS Debates 1937–46’, 179.

38 Daunton, ‘Payment and Participation’.

39
*Ibid*.

40 Nick Hayes and Barry Doyle, ‘Eggs, Rags and Whist Drives: Popular Munificence and the Development of Provincial Medical Voluntarism Between the Wars, *Historical Research*, 86, 712–40; Nick Hayes, ‘“Our Hospitals”? Voluntary Provision, Community and Civic Consciousness in Nottingham Before the NHS’, *Midland History*, 2012, 37, 84–105.

41
*Ibid*.

42 Hayes, ‘Our Hospitals?’, 94.

43 Helen McCarthy, ‘Associational Voluntarism in Interwar Britain’, in *The Ages of Voluntarism: How We Got to the Big Society* (Oxford: Oxford University Press, 2011), 47–68.

44 Gorsky, ‘Community Involvement in Hospital Governance in Britain’; Martin Gorsky, John Mohan and Tim Willis, *Mutualism and Health Care: Hospital Contributory Schemes in Twentieth-Century Britain* (Manchester: Manchester University Press, 2006), 124–44.

45 Mold, ‘Patient Groups and the Construction of the Patient-Consumer in Britain’.

46 Hall, ‘From Cottage to Community Hospitals’.

47 HC Deb 26 July 1946, vol 426, col. 402; Gorsky, Mohan and Willis, ‘Hospital Contributory Schemes and the NHS Debates 1937–46’.

48 Gorsky, Mohan and Willis, ‘Hospital Contributory Schemes and the NHS Debates 1937–46’; Christine Hogg, *Citizens, Consumers and the NHS: Capturing Voices* (London: Palgrave Macmillan, 2008).

49 For example, Mold, ‘Patient Groups and the Construction of the Patient-Consumer in Britain’; Alex Mold, *Making the Patient Consumer* (Manchester: Manchester University Press, 2015); Hogg, *Citizens, Consumers and the NHS*.

50 McConaghey, ‘The Evolution of the Cottage Hospital’.

51 Keith Atkins, ‘The Cottage Hospital Database’, in *The Cottage Hospital Movement in England & Wales 1850–1914*.

52 ‘Burgesses’ refers to elected or unelected officials.

53 ‘The Visit of H.R.H. Duke of Cambridge, K.G.’, 12 December 1898, S1(942.194)KIN, Kingston History Centre, Kingston-upon-Thames.

54 ‘Kingston Victoria Hospital: Report on Visit to Hospital’, 6 October 1930, A/KE/C/02/06/579, King Edward’s Hospital Fund for London, LMA, London.

55 ‘Kingston-upon Thames Victoria Hospital: Report of the Committee and Statement of Accounts for the Year 1928’, 1928, A/KE/C/02/06/579, King Edward’s Hospital Fund for London, London Metropolitan Archives (LMA), London.

56
*Ibid*.

57
*Ibid*.

58 ‘Review of Medical and Specialist Services’, 1949, LMA/4431/A/01/006, South West Metropolitan Regional Hospital Board, LMA, London.

59 ‘South West Metropolitan Regional Hospital Board: Report of the Medical Advisory Committee to the Board’, 21 July 1949, LMA/4431/A/01/007, South West Metropolitan Regional Hospital Board, LMA, London.

60 Foreign Letters: London, *The Journal of the American Medical Association*, 13 October 1951, 147, 681–82.

61 For example, ‘Report on Visit to Hospital’, 7 January 1948, DT35/168, General Nursing Council, TNA, London.

62 ‘Regional Board Rejects Plea for Victoria’, *Surrey Comet*, 8 October 1949, 5.

63 ‘Regional Board Rejects Plea for Victoria’, *Surrey Comet*; ‘Kingston and Malden Victoria Hospital’, 18 May 1950, A/KE/C/02/06/354, King Edward’s Hospital Fund for London, LMA, London.

64 ‘Shall Kingston Victoria Be Maternity Unit?’, *Surrey Comet*, 10 September 1949, 3.

65 ‘The Last Ditch: Doctors Dig in to Carry on Fight for Hospital’, *Surrey Comet*, 4 November 1950, 5.

66 ‘Doctors Seal Doors at Kingston Hospital’, *The Times*, 7 December 1950, 51868, 4.

67 ‘Hospital Defying Order to Close’, *The Times*, 1 November 1950, 51837, 3.

68
*Ibid*.; AP Archive: British Movietone, ‘Kingston Victoria Hospital Fight’, *YouTube* video, 3:41, 21 July 2015, https://www.youtube.com/watch?v=scWKj7yZEoc&t=20s&ab_channel=BritishMovietone.

69 ‘Kingston Council and Victoria’s Future’, *Surrey Comet*, 1 October 1949, 3.

70 Ralph G. Smith, ‘Hospital Threatened’, *Supplement to the BMJ*, 1950, 2, 251–54.

71 Stevens, *Medical Practice in Modern England*.

72 ‘Shall Kingston Victoria Be Maternity Unit?’, *Surrey Comet*, 10 September 1949, 3.

73
*Ibid.*

74 AP Archive: British Movietone, ‘Kingston Victoria Hospital Fight’.

75 HC Deb 30 January 1951, vol 483, col. 863.

76 ‘Hospital Defying Order to Close’, *The Times*, 1 November 1950, 51837, 3.

77 ‘Shall Kingston Victoria Be Maternity Unit?’, *Surrey Comet*.

78
*Ibid*.

79 ‘Review of Medical and Specialist Services’, 1949, LMA/4431/A/01/006, South West Metropolitan Regional Hospital Board, LMA, London.

80 ‘Shall Kingston Victoria Be Maternity Unit?’, *Surrey Comet*.

81 Hayes, ‘Did We Really Want a National Health Service?’.

82 For example, ‘Report on Visit to Hospital’, 7 January 1948, DT35/168, General Nursing Council, TNA, London.

83 ‘Report of the Finance & General Purposes Committee (Part I)’, 7 July 1948, LMA/4431/A/01/003, South West Metropolitan Regional Hospital Board, LMA, London.

84 ‘Report on Visit to Kingston Hospital’, May 1959, A/KE/H/01/02/094, King Edward’s Hospital Fund for London, LMA, London.

85 Hayes, ‘Did We Really Want a National Health Service?’, 643, 651.

86 HC Deb 30 January 1951, vol 483, col. 863–64.

87 ‘Kingston Council and Victoria’s Future’, *Surrey Comet*.

88 HC Deb 30 April 1946, vol 422, col. 44.

89 HL Deb 21 March 1944, vol 131.

90 HC Deb 26 July 1946, vol 426, col. 407.

91 ‘Shall Kingston Victoria Be Maternity Unit?’, *Surrey Comet*.

92 ‘Review of Medical and Specialist Services’, LMA/4431/A/01/006.

93 ‘Report on Visit to Hospital’, DT35/168.

94 AP Archive: British Movietone, ‘Kingston Victoria Hospital Fight’.

95 ‘Ministry Circular Supports Kingston’s Case’, *Surrey Comet*.

96 ‘Report of the Medical Advisory Committee to the Board’, August 1949, LMA/4431/A/01/007, South West Metropolitan Regional Hospital Board, LMA, London; ‘Ministry Circular Supports Kingston’s Case’, *Surrey Comet*.

97 ‘Kingston Council and Victoria’s Future’, *Surrey Comet*.

98 ‘Hospital Outcry Just a Political Move’, *Surrey Comet*, 29 October 1949, 5.

99 1945 Conservative Party General Election Manifesto, paragraph 6.

100 1950 Conservative Party General Election Manifesto, paragraph 81–82.

101 1950 Labour Party Manifesto.

102 HC Deb 30 January 1951, vol 483, col. 861.

103 ‘Kingston Group Hospital Management Committee Report’, October 1954, A/KE/C/02/06/280, King Edward’s Hospital Fund for London, LMA, London.

104 ‘Claremont Hospital Opened’, *Surrey Comet*, 2 June 1951, 5.

105 ‘Kingston Group Hospital Management Committee Report’, A/KE/C/02/06/280.

106 ‘No New Move in Victoria Fight’, *Surrey Comet*, 23 December 1950, 5.

107 ‘Report on Visit to Claremont Hospital, Surbiton’, 8 February 1952, DT33/1057, General Nursing Council, TNA, London.

108 ‘Kingston Group Hospital Management Committee Report’, A/KE/C/02/06/280.

109 ‘Conversion Upheld by Minister’, *The Times*, 3 March 1951, 51940, 3.

110 ‘£50,000 Appeal for New Victoria’, *Surrey Comet*; F. B. Lake, ‘Correspondence: New Voluntary Hospital for Kingston’, *British Medical Journal*, 1951, 2, 794.

111 ‘Correspondence: New Voluntary Hospital for Kingston’, *British Medical Journal*, 1951, 2, 794.

112 ‘New Hospital Outside State Service’, *The Times*, 29 May 1958, 54163, 14.

113 ‘New Victoria Foundation Magazine’, 1954, KX57, Kingston History Centre, Kingston-upon-Thames.

114 ‘Town’s Own Hospital Opened At Last’, *Surrey Comet*, 31 May 1958, 5.

115 ‘Still Deadlock’, *Surrey Comet,* 2 December 1950, 5.

116 Hayes, ‘Did We Really Want a National Health Service?’, 650.

117 ‘Hospital Inquiry Plea Again Rejected’, *Surrey Comet*, 3 February 1951, 5.

118 HC Deb 5 February 1951, vol 483, col. 1313.

119 HC Deb 5 February 1951, vol 483, col. 1313.

120 ‘Still Deadlock’, *Surrey Comet*.

121 ‘Hospital Controversy’, *The Times*, 24 November 1950, 51857, 4.

122 ‘Record Meeting Backs Victoria Fight’, *Surrey Comet*, 25 November 1950, 5.

123
*Ibid*.

124 ‘Record Meeting Backs Victoria Fight’, *Surrey Comet*, 25 November 1950, 5.

125 ‘Two Councils Act to Stop Victoria Scheme’, *Surrey Comet*, 15 October 1949, 3.

126 ‘The Last Ditch: Doctors Dig in to Carry on Fight for Hospital’, *Surrey Comet*, 4 November 1950, 5.

127 ‘Regional Board Rejects Plea for Victoria’, *Surrey Comet*, 8 October 1949, 5.

128 ‘New Moves in Fight for Hospital’, 26 November 1949, *Surrey Comet*, 5.

129 ‘Plea for Year’s Delay Rejected’, *Surrey Comet*, 11 November 1950, 5.

130 ‘Deadlock at Victoria’, *Surrey Comet*, 18 November 1950, 5.

131 ‘The Kingston Hospital Dispute: Mass Meeting of Protest’, *British Medical Journal*, 1950, 2, 224.

132 ‘MP Says Regional Board Abused Its Powers’, *Surrey Comet*, 25 March, 1950, 5.

133 David Stelling, ‘Letters to the Editor’, *The Spectator*, 23 February 1951, 18.

134 E. C. Warner, ‘Letter to the Editor: The Kingston Hospital Dispute’, *The Times*, 28 December 1950, 51884, 5.

135
*Ibid*.

136 HC Deb 26 July 1946, vol 426, col. 402.

137 ‘Doctors Fight to Retain the Hospital’, *Richmond Herald*, 18 November 1950, 12.

138
*Ibid*.

139 HC Deb 21 March 1950, vol 472, col. 1919–30.

140 ‘Flag Hauled Down’, *Surrey Comet*, 15 April 1950, 5.

141 ‘Still Deadlock’, *Surrey Comet*.

142 ‘Two Councils Act to Stop Victoria Scheme’, *Surrey Comet*.

143 ‘Violent Outcry Over Sacking of Opposition, *Surrey Comet*, 11 March 1950, 5.

144 ‘Hospital Board “Beheads” Opposition’, *Surrey Comet*, 4 March 1950, 5.

145
*Ibid*.

146 ‘Violent Outcry Over Sacking of Opposition’, *Surrey Comet*.

147 Daunton, ‘Payment and Participation’, 207.

148 Ministry of Health Circular R.H.B. (49) 143.

149 ‘Kingston Group Hospital Management Committee: Adjournment’, March 1950, MH88/157, South West Metropolitan Regional Hospital Board, TNA, London.

150 ‘Letter from RHB Chair to Ministry of Health’, March 1950, MH88/157, South West Metropolitan Regional Hospital Board, TNA, London.

151
*Ibid*.

152 ‘Hospital Management Committees: Appointment’, 1948–1952, MH88/157, South West Metropolitan Regional Hospital Board, TNA, London.

153
*Ibid*.

154 ‘Secret Debate by Committee’, *Surrey Comet*, 15 October 1949, 3.

155 HC Deb 7 December 1950 vol 482, col. 522.

156 HC Deb 21 March 1950, vol 472, col. 1919–30.

157 ‘Kingston Hospital Change: B.M.A. Proposes Inquiry’, *The Times*, 16 November, 51850, 4.

158 For example, ‘New Call for Hospital Inquiry’, *The Times*, 6 March 1951, 51942, 3.

159 For example, ‘Hospital Controversy’, *The Times*, 24 November, 51857, 4.

160 ‘MP says regional board abused its powers’, *Surrey Comet,* 25 March 1950, 5.

161
*Ibid*.

162 HC Deb 7 December 1950, vol 482, col. 522.

163 HC Deb 30 January 1951, vol 483, col. 868.

164 ‘Board to Receive Deputation’, *Surrey Comet*, 3 December 1949, 5.

165 ‘Kingston Victoria Hospital’, *The Times*, 15 February 1951, 51926, 3.

166 ‘Compromise Suggested by Regional Board’, *Surrey Comet*, 10 December 1949, 3.

167
*Ibid*.

168 ‘Fight to Save Hospital’, *Surrey Comet*, 11 February 1950, 5.

169 ‘Flag Hauled Down’, *Surrey Comet*.

170 ‘Kingston Hospital Conversion: Minister’s Decision’, *The Times*, 28 April 1951, 51987, 6.

171 ‘Medicine: Long Live the Vic!’, *Time*, 18 June 1951.

172 ‘Kingston and Malden Victoria Medical Foundation’, *The Times*, 18 October 1956, 53665, 1.

173 ‘Kingston and Malden Victoria Medical Foundation, Minutes of Annual General Meeting’, 26 October 1961, A/KE/C/02/06/354, King Edward’s Hospital Fund for London, LMA, London.

174 ‘New Victoria Foundation Magazine’, 1954, KX57, Kingston History Centre, Kingston-upon-Thames.

175 ‘Report on Visit to New Victoria Hospital’, 16 May 1960, New Victoria Hospital, Coombe Road, Kingston-Upon-Thames (1950–63), A/KE/C/02/06/354, King Edward’s Hospital Fund for London, LMA, London.

176 ‘New Victoria Foundation Magazine’, KX57.

177 ‘Correspondence: New Voluntary Hospital for Kingston’, *British Medical Journal.*

178 Warner, ‘Letter to the Editor: The Kingston Hospital Dispute’, *The Times*.

179 ‘Medical Notes in Parliament: Kingston Dispute’, *Supplement to the British Medical Journal*, 1950, 2, 245–50; Warner, ‘Letter to the Editor: The Kingston Hospital Dispute’, *The Times*; ‘Doctors’ Plan at Kingston’, *The Times*, 2 June 1951, 52017, 4; ‘Doctors and Hospitals’, *The Times*, 2 June 1952, 52327, 7.

180 ‘Conversion Upheld By Minister’, *The Times*.

181 ‘The Visit of H.R.H Duke of Cambridge, K.G.’, S1(942.194)KIN.

182 HC Deb 21 June 1950, vol 476, col. 1437.

183 ‘Medical News: Kingston Victoria Hospital’, *British Medical Journal*, 1951, 1, 1028.

184 ‘Town’s Own Hospital Opened at Last’, *Surrey Comet*, 31 May 1958, 5.

185 ‘The New National Health Service’, 1948, WA500 1948G78n, Wellcome Collection, London.

186 Seaton, ‘Against the Sacred Cow’, 425.

187 ‘£50,000 Appeal for New Victoria’, *Surrey Comet*.

188 ‘Kingston Victoria Hospital’, *Supplement to the British Medical Journal.*

189 ‘£50,000 Appeal for New Victoria’, *Surrey Comet*.

190
*Ibid*.

191 ‘New Voluntary Hospital’, *The Times*, 23 October 1954, 53070, 3.

192 ‘The New Victoria Hospital, Kingston, Annual General Meeting’, 26 October 1961, MS-HALER/312/14, Royal College of Physicians, London, 3–4.

193 ‘New Victoria Foundation Leaflet’, c.1958, KT110/4/4, Kingston History Centre, Kingston-upon-Thames.

194 ‘£50,000 Appeal for New Victoria’, *Surrey Comet*.

195 New Hospital Outside State Service, *The Times*, 29 May 1958, 54163, 14.

196 ‘Letter from King’s Fund to New Victoria’, A/KE/C/02/06/354.

197 ‘Letter from King’s Fund to Sir Archibald Gray’, 16 January 1959, A/KE/C/02/06/354, King Edward’s Hospital Fund for London, LMA, London.

198
*Ibid*.

199 ‘Letter from New Victoria General Secretary to King’s Fund Secretary’, 9 September 1960, A/KE/C/02/06/354, King Edward’s Hospital Fund for London, LMA, London.

200 ‘Letter from King’s Fund Deputy Secretary to New Victoria’, 30 November 1962, A/KE/C/02/06/354, King Edward’s Hospital Fund for London, LMA, London.

201 ‘Letter from Kenneth Robson to King's Fund Assistant Secretary', 20 May 1962, A/KE/C/02/06/354, King Edward's Hospital Fund for London, LMA, London.

202 Medical News: Kingston New Victoria Hospital, *British Medical Journal*, 1966, 2, 1337.

203 Medical News: New Victoria Hospital, Surrey, *British Medical Journal*, 1973, 2, 724–25.

204 Nicholas Deakin and Justin Davis Smith, ‘Labour, Charity and Voluntary Action: The Myth of Hostility’, in *The Ages of Voluntarism: How We Got to the Big Society* (Oxford: Oxford University Press, 2011), 69–93.

205 Harris and Creswell, ‘The Legacy of Voluntarism’.

206 Stefan Ramsden and Rosemary Cresswell, ‘First Aid and Voluntarism in England, 1945–85’, *Twentieth Century British History*, 2019, 30, 504–30.

207 ‘Labour Attack on New Victoria Hospital’, *Surrey Comet*, 7 June 1958, 17.

208 ‘Medicine: Long Live the Vic!’, *Time*.

209 Hayes, ‘Did We Really Want a National Health Service?’.

210 HC Deb 26 July 1946, vol 426, col. 402.

211 Warner, ‘Letter to the Editor: The Kingston Hospital Dispute’, *The Times*.

212 ‘Correspondence: New Voluntary Hospital for Kingston’, *British Medical Journal.*

213 ‘Hospital Management Committees: Appointment’, 1948–1952, MH88/157, South West Metropolitan Regional Hospital Board, TNA, London.

## Notes

Dr Steph Haydon is a Research Fellow at the University of Birmingham’s Third Sector Research Centre. She is currently working on the ‘*Border Crossings: Charity and voluntarism in Britain’s mixed economy of health care since 1948*’ project. Her research explores charity and philanthropy, particularly high-net-worth philanthropy and the interactions between charity and the state.

